# Prediction of Viscoelastic Properties of Enzymatically Crosslinkable Tyramine–Modified Hyaluronic Acid Solutions Using a Dynamic Monte Carlo Kinetic Approach

**DOI:** 10.3390/ijms22147317

**Published:** 2021-07-07

**Authors:** Filippos F. Karageorgos, Costas Kiparissides

**Affiliations:** 1 Chemical Process & Energy Resources Institute, 6th Km Harilaou-Thermi Rd., P.O. Box 60361, 57001 Thessaloniki, Greece; karageof@certh.gr; 2 Department of Chemical Engineering, Aristotle University of Thessaloniki, 54124 Thessaloniki, Greece

**Keywords:** Monte Carlo simulation, crosslinking kinetic modeling, enzyme mediated crosslinking, gelation, hydrogel molecular properties, viscoelastic properties, tyramine-modified hyaluronic acid crosslinking, polymerization

## Abstract

The present study deals with the mathematical modeling of crosslinking kinetics of polymer–phenol conjugates mediated by the Horseradish Peroxidase (HRP)-hydrogen peroxide (H_2_O_2_) initiation system. More specifically, a dynamic Monte Carlo (MC) kinetic model is developed to quantify the effects of crosslinking conditions (i.e., polymer concentration, degree of phenol substitution and HRP and H_2_O_2_ concentrations) on the gelation onset time; evolution of molecular weight distribution and number and weight average molecular weights of the crosslinkable polymer chains and gel fraction. It is shown that the MC kinetic model can faithfully describe the crosslinking kinetics of a finite sample of crosslinkable polymer chains with time, providing detailed molecular information for the crosslinkable system before and after the gelation point. The MC model is validated using experimental measurements on the crosslinking of a tyramine modified Hyaluronic Acid (HA-Tyr) polymer solution reported in the literature. Based on the rubber elasticity theory and the MC results, the dynamic evolution of hydrogel viscoelastic and molecular properties (i.e., number average molecular weight between crosslinks, *M_c_*, and hydrogel mesh size, *ξ*) are calculated.

## 1. Introduction

Hydrogels are used in many biomedical applications, including drug delivery systems, regenerative tissue engineering, contact lenses, etc. Hydrogels are three-dimensional (3D) networks that can be formed via the crosslinking of hydrophilic polymers. The crosslinking of polymer chains can be effected by the use of a suitable crosslinking agent, leading to the formation of either chemical (i.e., covalent, etc.) or physical (i.e., chain entanglements, hydrophobic interactions, etc.) crosslinks and, finally, to a 3D network. Hydrogels are biocompatible materials with the ability to swell via the absorption of a large amount of water. The polymer type (i.e., chemical nature of structural monomer units, molecular weight, presence of functional groups, etc.) and synthesis conditions (i.e., polymer and crosslinker concentrations, pH, temperature, etc.) determine the final molecular, physical and mechanical properties of a hydrogel, including the number of crosslinks, degree of swelling, hydrogel degradation kinetics, viscoelastic properties, etc.

Physically crosslinked hydrogels are commonly soft materials and, thus, not suitable for biomedical applications in which the hydrogels undergo high compressive and shear stresses. On the other hand, chemically crosslinked hydrogels are stiff materials and can withstand high stresses. Thus, they can be used in tissue engineering applications, implantable devices, etc. A problem that often arises in chemically crosslinked hydrogels is their high cytotoxicity caused by the presence of residual chemical crosslinker. For this reason, in many applications, the crosslinking of the polymer chains is carried out in the presence of an enzyme.

An important biomedical application of hydrogels is that of injectable hydrogels. Injectable hydrogels are formed via the co-injection of a polymer solution with a suitable crosslinker to the site of interest, where they undergo a fast gelling process. The so-formed site-specific hydrogel can be used as a local drug delivery device for the controlled release of an active pharmaceutical ingredient (API) or as a scaffold for cell proliferation and tissue regeneration.

Horseradish Peroxidase (HRP) has been commonly used in the enzymatically catalyzed crosslinking of polymer–phenol conjugates, for it offers good control of the final hydrogel properties [1]. HRP has been used in the synthesis of injectable hydrogels, microcapsules [2], gel sheets [3], bone cement [4], hollow fibers [5], etc. In Table 1, a selected list of publications on the enzymatic crosslinking of various polymer–phenol conjugates in the presence of the HRP/H_2_O_2_ catalytic system are reported.

The mathematical modeling of crosslinking reactions is a subject that has attracted the wide interest of the polymer research community since the pioneering work of Flory in 1941 [15,16]. Since then, many publications and monographs have appeared in the open literature [17,18,19,20]. However, despite the large number of publications on the mathematical modeling of polymer crosslinking, there is only a very limited number of papers dealing with the modeling of polymer–phenol conjugates crosslinking in the presence of the HRP/H_2_O_2_ initiation system. An example of modeling of phenolic polymerization catalyzed by HRP can be found in the work of Ryu and coworkers [21]. However, the postulated kinetic mechanism did not include an enzyme deactivation reaction. In the publication of Yamagishi et al. [22], the Monte Carlo simulation of phenol–formaldehyde networks was described. In a recent publication by Kiparissides et al. [23], a comprehensive kinetic model and the method moments were employed to predict the onset gelation time of a tyramine modified Hyaluronic Acid (HA-Tyr) aqueous solution in terms of the HRP and H_2_O_2_ concentrations.

With regards to the modelling and prediction of time-varying viscoelastic and swelling properties of enzymatically crosslinked polymer–phenol conjugates, there are not any publications in the open literature. In a recent publication [23], the importance of accurate prediction of the viscoelastic properties, flowability, spreading, adhesion and stability of a crosslinkable polymer solution deposited onto the nasal olfactory site was elaborated. In drug delivery applications, the viscoelastic and swelling properties of a hydrogel are important, since they determine the release rate of the active pharmaceutical ingredient (API) from the hydrogel matrix. In tissue engineering, hydrogels are employed as tissue mimics of the extracellular matrix (ECM), providing a robust platform for cell cultures [24]. Note that the ECM is known to transmit external mechanical loads to resident cells in order to modulate the cell behaviors, including cell spreading, proliferation, migration and differentiation [24,25]. Depending on the polymer type (i.e., chemical nature of structural monomer units, molecular weight, presence of functional groups, etc.) and synthesis conditions (i.e., polymer and crosslinker concentrations, temperature, etc.), hydrogels having tunable mechanical properties, desired porosity and a water uptake capacity can be synthesized. Thus, it is of crucial importance the model-based prediction of viscoelastic and swelling properties of hydrogels in terms of the material and synthesis conditions and, thus, the optimal selection and design of a hydrogel for a biomedical application.

In the present work, a Monte Carlo modeling approach is described to quantify the effects of crosslinking conditions (i.e., polymer molecular weight and concentration, degree of phenol substitution and HRP and H_2_O_2_ concentrations) on the gelation onset time; evolution of gel fraction and molecular weight distribution of the crosslinked polymer chains and viscoelastic hydrogel properties. A multidimensional dynamic Monte Carlo model, based on the Gillespie’s original algorithm [26,27], is developed to calculate the dynamic evolution of molecular properties (i.e., number and weight average molecular weights, gel fraction, number of crosslinks, etc.) in a finite sample of polymer chains undergoing enzymatic crosslinking. In the MC model, each polymer chain is characterized by four internal variables—namely, the polymer chain length, i.e., degree of polymerization, the residual phenol content in the reactive polymer chains, the number of the activated phenol groups and the number of crosslinks. Note that the MC model can also provide information on the evolution of molecular weight distribution of polymer chains before and after the gelation point. The derived model is first validated using experimental kinetic measurements on a polymer–phenol conjugate system [8] (i.e., tyramine modified Hyaluronic Acid (HA-Tyr)). It is shown that the model can accurately predict the gelation onset time of the crosslinkable system in terms of the HRP and H_2_O_2_ concentrations. Based on the MC-calculated hydrogel molecular properties (i.e., number of crosslinks in the hydrogel network, gel fraction, etc.), the viscoelastic properties (i.e., the storage modulus, *G′*), time required for the storage modulus to reach a plateau value and the polymer volume fraction in the swollen state, *u_2,s_*, are calculated for a tyramine modified Hyaluronic Acid (HA-Tyr) crosslinkable polymer solution [8]. It is shown that model predictions are in excellent agreement with the experimental measurements of Lee et al. (2008) [8] for the HA-Tyr system.

In what follows, the enzymatic crosslinking mechanism for polymer–phenol conjugates in the presence of the HRP/H_2_O_2_ initiation system is postulated. In Section 3, a detailed description of the stochastic MC kinetic crosslinking model is presented. In Section 4, the derived dynamic MC model is validated using the experimental results of Lee [8] for the tyramine modified Hyaluronic Acid (HA-Tyr) system. The effects of the HPR and H_2_O_2_ concentrations on the gelation onset time and the average molecular and viscoelastic properties of the synthesized hydrogels are assessed, and the model predictions are successfully compared with reported experimental measurements [8]. Moreover, predictions of the hydrogel molecular properties (i.e., average molecular weight between crosslinks, *M_c_*, and hydrogel mesh size, *ξ*) are presented. Finally, in Section 5, the main conclusions of the present work are summarized.

## 2. Enzymatic Crosslinking of Polymer–Phenol Conjugates

### 2.1. The Postulated Kinetic Mechanism

Horseradish Peroxidase or HRP is an enzyme extracted from the roots of horseradish (Armoracia rusticana) [28,29]. It consists of 308 amino acid residues, four disulfide bridges between cysteine residues a heme group and two calcium atoms [30,31]. The heme group is a complex of Fe(III) and protoporphyrin. HRP can oxidize organic and inorganic compounds using H_2_O_2_ [28].

**Figure 1:**
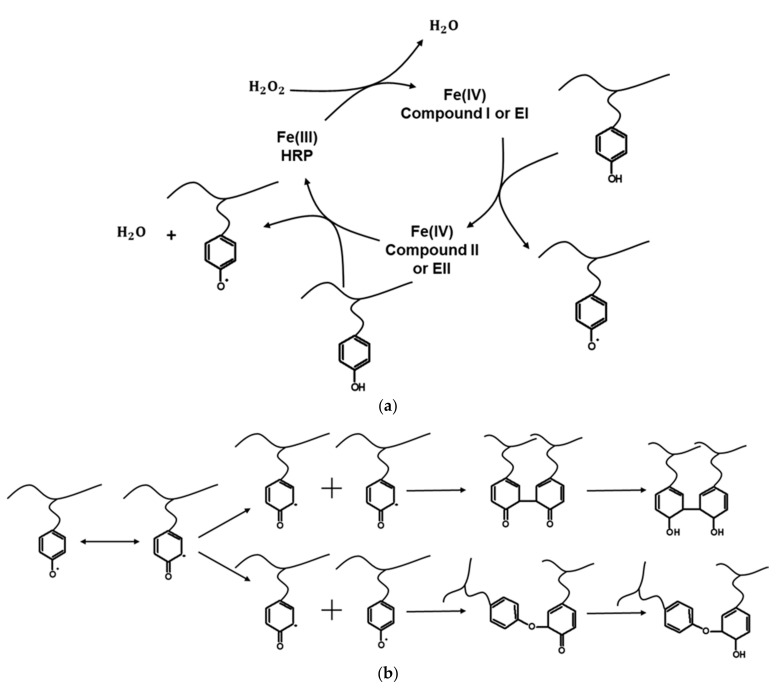
(**a**) Activation of polymer–phenol conjugates in the presence of the HRP enzyme and hydrogen peroxide. (**b**) Isomerization of phenolic radicals and crosslinking reactions between different types of “live” polymer chains and enolization of crosslinked polymer chains (adapted from reference [23]).

The HRP-catalytic cycle starts with the heme being in the resting state, Fe(III) (Figure 1). In the presence of hydrogen peroxide (i.e., oxidizing substrate), the HRP is oxidized to compound I (EI), which is two oxidation states above the resting state, including a Fe(IV) = O ferryl group and a porphyrin cation radical, with the simultaneous formation of one molecule of H_2_O. Subsequently, the polymer–phenol conjugate is activated by the catalytic action of compound I, resulting in the formation of compound II (EII), which includes the neutralized porphyrin, Fe(IV) = O, and a phenol radical in the polymer chain (see Figure 1). The HRP-catalytic cycle returns to its initial state, Fe(III), via the reduction of compound II by the polymer–phenol conjugate, with the simultaneous formation of a phenol radical in a polymer chain and a water molecule [29,30].

There are also two other forms of HRP known as compounds III and IV. Compound III is an inactive but reversible form of HRP. Compound III can be formed via the reaction of Compound II with hydrogen peroxide when the second is in excess. Compound IV is an irreversibly inactive form of HRP. Compound IV is formed from Compound III or Compound I when the hydrogen peroxide concentration is in excess. It is worth mentioning that the enzyme deactivation mechanism has not been fully elucidated [32].

Note that the phenolic radicals can also undergo an isomerization reaction [33] (Figure 1b). The generated “live” polymer chains can undergo a crosslinking reaction via the phenolic radical groups shown in Figure 1a. In particular, two different types of crosslinks can be formed—namely, a C-C linkage between ortho-carbons (a more common one) and a C-O linkage between an ortho-carbon and the phenolic oxygen. The latter linkage is a less common one. After the crosslinking of “live” polymer chains, an enolization reaction can take place [33] (Figure 1b).

In accordance with the postulated kinetic mechanism for the enzymatic crosslinking of the polymer–phenol conjugates described above, the following elementary reactions were considered to detail the crosslinking kinetic developments of the phenol modified polymer chains (i.e., tyramine modified Hyaluronic Acid (HA-Tyr)): (i) activation of the HRP enzyme by hydrogen peroxide (Equation (1), (ii) generation of “live” polymer chains via the activation of phenol rings (Equations (2) and (3)), (iii) enzyme deactivation via the reaction of compound II with hydrogen peroxide (Equation (4)), (iv) termination by combination of “live” polymer chains (Equation (5)) and (v) internal cyclization reaction (Equation (6)). 

Initiation/Activation/Deactivation Reactions

(1) $$E+H_{2}O_{2}\overset{k_{1}}{\to }E_{I}$$

(2) $$E_{II}+R_{x,m,a,c}\overset{k_{2}}{\to }R_{x,m-1,a+1,c}+E_{II}$$

(3) $$E_{II}+R_{x,m,a,c}\overset{k_{3}}{\to }R_{x,m-1,a+1,c}+E$$

(4) $$E_{II}+2H_{2}O_{2}\overset{k_{4}}{\to }E_{IV}$$

Termination by Combination

(5) $$R_{x,m,a,c}+R_{y,n,b,d}\overset{k_{5}}{\to }R_{x+y,m+n,a+b-2,c+d+1}$$

Polymer Chain Cyclization

(6) $$R_{x,m,a,c}\overset{k_{6}}{\to }R_{x,m,a-2,c+1}$$

The symbol $R_{x,m,a,c}$ denotes a polymer chain having *x* monomer units, *m* unreacted phenol groups, *a* activated phenol groups and *c* crosslinks. The cyclization reaction (Equation (6)) is considered to take place only in the gel phase (i.e., after the gelation onset time). In the present work, the cyclization reaction was treated as an intramolecular biomolecular reaction between the activated phenol groups in the gel phase [34,35]. Note that the largest molecule in the MC simulated crosslinkable system is taken as the gel phase [36].

Moreover, it was assumed that the reaction leading to the formation of the inactive form of HRP (i.e., compound IV) exhibits a second-order dependence on hydrogen peroxide. To further simplify the present kinetic model developments, the reaction steps related to the isomerization of phenol radicals and enolization of the crosslinked polymer chains were not accounted for. Note that the present kinetic model accounts for the total number of crosslinks; that is, no distinction between the C-C and C-O crosslinks was made.

### 2.2. The Stochastic Monte Carlo Approach

Stochastic Monte Carlo (MC) methods make use of random numbers to select an event (e.g., reaction step) from a set of possible outcomes (e.g., reaction mechanism). MC methods can be applied to a wide variety of natural sciences and engineering problems. Among others, they have found application in polymer science and, in particular, to the simulation of polymer chain microstructural details that are difficult or even impossible to predict with other deterministic modeling techniques. This specific modeling ability is the main advantage of the stochastic MC methods and their wide application to polymer science and reaction engineering [37].

The two main MC approaches that are currently used for the stochastic simulation of the polymerization kinetics are based on the pioneering works of Gillespie [26] and Tobita [38]. Thus, the vast majority of stochastic simulation studies in polymerization are based on these two approaches. The Gillespie approach, known as the stochastic simulation algorithm (SSA), is an exact method that is used to simulate the kinetics of a spatially homogeneous chemical reaction system on the basis of the reaction probability density function [26,27]. It is known that the Gillespie algorithm is fully equivalent to the master-equation approach [26]. In spite of the fact that the master-equation approach is exact and elegant; it is usually not very useful for making practical numerical calculations [27]. Thus, for complex reactive systems (i.e., like the present multidimensional crosslinking system), the Gillespie algorithm is a more efficient and easier to apply method. The SSA is an event driven stochastic method in which different events (i.e., chemical reactions) take place according to the estimated instantaneous probabilities of the relevant chemical reactions while the duration of each time-step, Δ*t*, is calculated on the basis of the cumulative reaction rate of the reactive system [39].

(7) $$\Delta t={\left(\overset{N_{RE}}{\underset{j=1}{\sum }}R_{j}\right)}^{-1}\ln\left(\frac{1}{rand1}\right)$$

In the above expression, *rand*1 is a randomly generated number from a uniform distribution in the range of [0,1], and *R_j_* denotes the stochastic reaction rate in s^−1^ of the chemical reaction “*j*”*,* calculated in terms of the corresponding kinetic rate constant, *k_j,MC_*, in *(s)^−^*^1^ and the total number of potential combinations of the molecules involved in the randomly selected reaction step, $X_{c}$ [39,40].

(8) $$R_{j}=k_{j,MC}X_{c};\;\;\;\;\;\;\;\;\;j=1,2,3,\ldots ,N_{RE}$$

*N_RE_* is the number of distinct chemical reaction steps in the system.

Let us assume a homogenous chemical system of volume *V* containing *N_s_* different species *S_i_* (*i* = 1, 2, 3, …, *N_s_*). Assume that *X_i_* denotes the number of molecules of species “*i*” in the system. Following the original *MC* developments of Gillespie [26], the reaction rate for a unimolecular chemical reaction,$\;S_{m}\overset{k_{m,MC}}{\to }S_{o}$, will be given by

(9) $$R_{m}=k_{m,MC}X_{m};\;\;\;\;\;\;\;\;\;k_{m,MC}=k_{m}$$

where *k_m,MC_* is the stochastic kinetic rate constant of the reaction, and $k_{m}$ is the respective experimental/deterministic value of the kinetic rate constant [41]. Note that, for unimolecular reactions, the numerical values of the stochastic and experimentally measured kinetic rate constants will be identical.

Accordingly, for the bimolecular reaction between two different species, *S_m_* and *S_n_*, $S_{m}+S_{n}\overset{k_{mn,MC}}{\to }S_{o}$, the stochastic reaction rate will be given by

(10) $$R_{mn}=k_{mn,MC}X_{m}X_{n};\;\;\;\;\;\;\;\;\;k_{mn,MC}=k_{mn}/\left(VN_{A}\right)$$

where *k_mn,MC_* denotes the *MC* kinetic rate constant of the bimolecular reaction, $k_{mn}$ is the respective experimentally observed value of the kinetic rate constant [41] and *N_A_* is the Avogadro’s number.

Note that, for a bimolecular reaction between the same species *S_m_*, $S_{m}+S_{m}\overset{k_{mm,MC}}{\to }S_{o}$, the stochastic reaction rate will be given by

(11) $$R_{mm}=\left(1/2\right)k_{mm,MC}X_{m}\left(X_{m}-1\right);\;k_{mm,MC}=2k_{mm}/\left(VN_{A}\right)$$

where *k_mm,MC_* and $k_{mm}$ denote the stochastic and experimental kinetic rate constants of the reaction, respectively [41].

For the implementation of the Gillespie’s direct MC method, two random numbers (i.e., *rand*1 and *rand*2) are selected that are subsequently used to identify the chemical reaction that will next occur (*rand*2) and the time step Δ*t* between two consecutive reaction events (*rand*1). The *rand*1 and *rand*2 are randomly generated numbers uniformly distributed in the range of 0 to 1.

To identify the reaction that will take place at a given time instant, the following relation is employed:

(12) $$\overset{j-1}{\underset{i=1}{\sum }}P_{i}<rand2\leq \overset{j}{\underset{i=1}{\sum }}P_{i}$$

*P_i_* is the probability of occurrence of reaction “*i*” and is calculated by

(13) $$P_{i}=R_{i}/\overset{N_{RE}}{\underset{j=1}{\sum }}R_{j}$$

**Figure 2:**
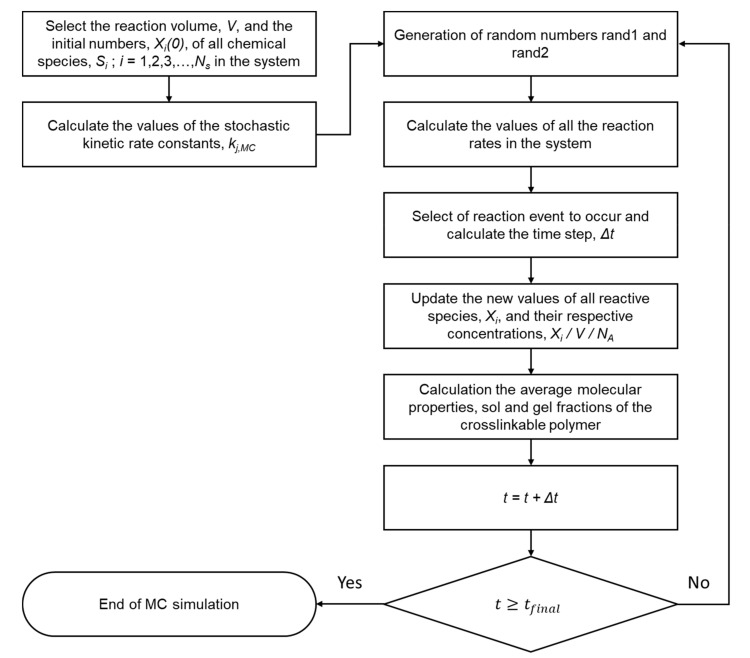
Schematic representation of Gillespie’s direct method implementation.

In Figure 2, a flow diagram depicting the implementation of Gillespie’s direct *MC* method to the enzymatic crosslinking of polymer–phenol conjugates is depicted.

### 2.3. Development of a 4D MC Kinetic Crosslinking Model

Following the original developments of Gillespie’s MC direct method, a four-dimensional Monte Carlo model was developed to describe the crosslinking of tyramine modified Hyaluronic Acid (HA-Tyr) in the presence of the HRP/H_2_O_2_ oxidation system. In Table 2, the derived stochastic reaction rates based on the postulated kinetic mechanism (Equations (1)–(6)) are reported for the 4D MC kinetic model. To simplify the MC developments and reduce the computational time from several days to approximately one day per single MC run, it was assumed that the reaction mixture was spatially homogeneous, and the diffusional limitations were negligible.

$N_{E}$, $N_{H_{2}O_{2}\;}\;\mathrm{and}\;N_{R}$ are the total number of the enzyme, hydrogen peroxide molecules and polymer chains, respectively. The symbol $R_{x_{i},m_{i},a_{i},c_{i}}$ denotes the “*i*” polymer chain with *x* monomeric units, *m* residual phenol groups, *a* activated phenol groups and *c* crosslinks. $G_{x,m,a,c}$ is the largest polymer chain, having *x* monomeric units, *m* residual phenol groups, *a* activated phenol groups and *c* crosslinks.

Note that Reaction (4) is a trimolecular reaction. In Reaction (5), the kinetic rate constant is multiplied by 0.5 to take into account that the number of possible combinations of two terminating polymer chains of lengths, *x-y* and *y* (i.e., to obtain a polymer chain of length *x*), is counted twice [42]. Therefore, for termination by combination, the total number of unique combinations of *N_R_* polymer chains will be [42]

(14) $$N_{c}=\frac{1}{2}\overset{N_{R}-1}{\underset{i=1}{\sum }}\left(a_{i}\cdot R_{x_{i},m_{i},a_{i},c_{i}}\overset{N_{R}}{\underset{l=i+1}{\sum }}a_{l}\cdot R_{x_{l},m_{l},a_{l},c_{l}}\right)$$

From the implementation of the above-described dynamic MC algorithm (see Figure 2 and Table 2), one can calculate the time evolution of the crosslinkable HA-Tyr system, namely, the gelation onset time, the concentration of residual phenol groups, the concentration of activated phenol groups, the number of crosslinks per initial polymer chain and the sol and gel fractions. In addition, the 4D MC algorithm provides detailed information on the Number Chain Length Distribution (NCLD) and the bivariate Chain Length–Number of Crosslinks Distribution, as well as the dynamic evolution of the number and weight average molecular weights (i.e., *M_n_* and *M_w_*) in the crosslinkable system. 

In particular, the number and weight average molecular weights will be given by the following equation [40,43]:

(15) $$M_{n}=\frac{\sum_{i=1}^{N_{R}}M_{i}}{N_{R}};\;M_{w}=\frac{\sum_{i=1}^{N_{R}}M_{i}^{2}}{\sum_{i=1}^{N_{R}}M_{i}}$$

where *M_i_* (=*x_i_MW_m_*) represents the molecular weight of the “*i*th” polymer chain, *x_i_* denotes the degree of polymerization of the “*i*th” polymer chain, *N_R_* is the total number of polymer chains in the reactive system and *MW_m_* is the molecular weight of the repeating structural unit.

## 3. Prediction of Viscoelastic Properties of a Crosslinkable Polymer Solution

In what follows, the connection of the MC simulation results with the hydrogel viscoelastic and other molecular structural properties (e.g., *M_c_* and *ξ*) is outlined. It is well-known that the hydrogel synthesis conditions (i.e., polymer, HRP and H_2_O_2_ concentrations; degree of HA functionalization, etc.) do affect the final physical and viscoelastic properties of the hydrogel. On the other hand, the fundamental theory of rubber elasticity can provide a sound base for the calculation of hydrogel viscoelastic properties in terms of the molecular structure of the synthesized 3D hydrogel network. 

**Figure 3:**
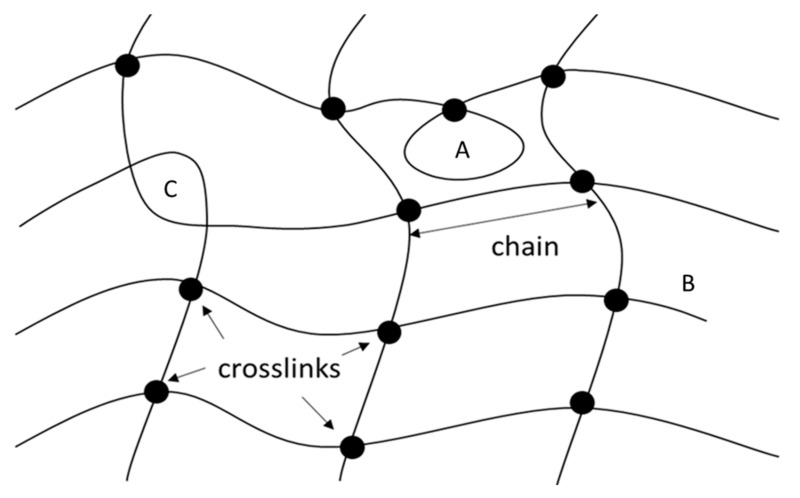
Schematic representation of a hydrogel network: (**A**) intramolecular loop, (**B**) free polymer chain ends and (**C**) polymer chain entanglement.

In particular, the relaxation modulus (*G*) of the hydrogel can be calculated in terms of the MC calculated concentration of crosslinks, *v_c_*. Note that the relaxation modulus, *G(t)*, of a crosslinked network, at long times, approaches a nearly constant value, *G_e_*, representing the equilibrium shear modulus, as described by the rubber elasticity theory [44]. Moreover, the storage modulus of a crosslinkable system, *G′(ω)*, at low frequencies, approaches the same characteristic value of *G_e_* [44]. According to the fundamental work of Treolar [45] on rubber elasticity, the equilibrium shear modulus of an ideal rubber elastic network can be calculated by the following equation:

(16) $$G_{e}=vkT=2v_{o}kT=2v_{c}RT=\frac{\rho }{M_{c}}RT$$

where *v* is the number of network polymer chains or segments per unit volume (1/m^3^). For the case of normal crosslinking (in which four polymer chains meet at each junction point; see Figure 3), *v* will be equal to twice the number of crosslinks per unit volume, *v_o._*, (1/m^3^). *k* is the Boltzmann’s constant (J/K), *T* is the absolute temperature (K) and *v_c_* is the crosslinks concentration (mol/m^3^). *R* is the universal gas constant (J∙mol^−1^∙K^−1^), *ρ* is the density of the polymer and *M_c_* is the number average molecular weight between two crosslinks [45].

In reality, 3D polymer networks are not ideal and include a number of defects. Flory [46] mentioned three types of defects—namely, chain intramolecular crosslinks, chain free or loose ends and chain entanglements (see Figure 3). The term intramolecular crosslinks refers to the crosslinking of two functional groups residing on the same polymer chain, resulting in the formation of a chain loop. In the case that this chain loop has no other intervening crosslinks with other chains in the network, then the loop will not provide any elastic contribution to potential network deformations. The term chain free or loose end refers to chains (i.e., chain segments) having only one of the two ends connected to the network by a crosslink. As a result, this free chain segment does not provide any elastic contribution to the network’s elastic modulus. Finally, the term chain entanglement refers to the reticular or spherical structure formed by the entanglement points formed in a polymer chain or between polymer chains, which limits the polymer chains mobility. This nonchemical connection can be equivalent to a crosslink and, thus, potentially provide an elastic contribution to the 3D polymer network.

From the above three cited network defects, Flory treated the case of free end polymer chains connected to a network. Assuming that *N_p_* is the number of original or primary molecules before crosslinking, Flory argued that *N_p_*-1 intermolecular linkages are required to link the primary molecules together into a single ramified structure in which there are no closed loops. After this point, each additional crosslink will produce one closed loop or two network chains. Moreover, Flory argued that only these additional crosslinks are effective in the network formation [45,46]. Thus, for the calculation of $G_{e},\;$the following equation can be used:

(17) $$G_{e}=v_{e}kT=2v_{o}\left(1-\frac{N_{p}}{v_{o}}\right)kT=2v_{c}\left(1-\frac{\rho }{v_{c}M_{n}}\right)RT=\frac{\rho }{M_{c}}\left(1-\frac{2M_{c}}{M_{n}}\right)RT$$

where $v_{e}$ is the number of effective chains per unit volume (m^−3^), $N_{p}$ is the number of primary molecules before crosslinking (m^−3^) and$\;M_{c}$ is the number average molecular weight between two crosslinks for an ideal network [45].

The number of primary molecules can be calculated in terms of the polymer density, *ρ*, the Avogadro’s number, *N_A_*, and the number average molecular weight of the primary molecules, *M_n_*.

(18) $$N_{p}=\rho N_{A}/M_{n}$$

Accordingly, the total number of crosslinks, $v_{o},$ per unit volume will be given by

(19) $$2v_{o}=\rho N_{A}/M_{c}$$

A more general form of Equation (16) that takes into account the above three referred network chain defects, as well as other effects (e.g., network crosslinks fluctuations), was presented by Ferry [44]:

(20) $$G_{e}=g\left(\frac{\bar{r_{E}^{2}}}{\bar{r_{0}^{2}}}\right)vkT=g\left(\frac{\bar{r_{E}^{2}}}{\bar{r_{0}^{2}}}\right)2v_{o}kT=g\left(\frac{\bar{r_{E}^{2}}}{\bar{r_{0}^{2}}}\right)2v_{c}RT=g\left(\frac{\bar{r_{E}^{2}}}{\bar{r_{0}^{2}}}\right)\frac{\rho }{M_{c}}RT$$

where *g* is a correction parameter to account for network crosslinks that are mobile and can experience fluctuations. For the case of tetrafunctional crosslinking, Ferry reported a value for g equal to ½ [44]. Other authors treated the *g* parameter as a correction factor for permanent network chain entanglements [46,47] and intramolecular crosslinks that do not contribute to the network’s stiffness [47]. The numerical value of the ratio ($\bar{r_{E}^{2}}/\bar{r_{0}^{2}}$) is normally close to unity. $\bar{r_{E}^{2}}$ is the mean square end-to-end distance of a strand, and $\bar{r_{0}^{2}}$ is the mean square end-to-end distance of a strand of the same length, assuming that the strand is not constrained by the presence of crosslinks.

From Equations (17) and (20), one can obtain the following equation for *G_e_* for a nonideal network:

(21) $$G_{e}=gv_{e}kT=g2v_{o}\left(1-\frac{N_{p}}{v_{o}}\right)kT=g2v_{c}\left(1-\frac{\rho }{v_{c}M_{n}}\right)RT=g\frac{\rho }{M_{c}}\left(1-\frac{2M_{c}}{M_{n}}\right)RT$$

It should be pointed out that Equations (16)–(21) are applicable to dry polymer networks. Thus, in the case of crosslinkable polymer solutions, the polymer density, *ρ*, in the above equations should be replaced by the polymer concentration of the crosslinkable solution, *C* [48,49]. Note that using available experimental measurements of *G_e_* and Equations (16), (17), (20) and (21), the average molecular weight between two crosslinks, *M_c_*, can be calculated.

In the present work, the value of *G_e_* was calculated from Equation (21) in terms of the concentration of crosslinks, *v_c_* (mol/m^3^), obtained from the solution of the 4D Monte Carlo kinetic model describing the enzymatic crosslinking of the HA-Tyr polymer solution in the presence of the HRP/H_2_O_2_ oxidation system. Moreover, the average molecular weight between two crosslinks, *M_c_*, will be given by the following equation:

(22) $$M_{c}=C/\left(2\cdot v_{c}\right)$$

where *C* is the concentration of polymer chains in the solution.

Regarding the calculation of the polymer volume fraction at the equilibrium swollen state, $u_{2,s}$, several equations have been proposed in the literature, including the pioneering work of Flory [50], who related the value of $u_{2,s}$ with *M_c_*. Peppas and Merrill [51] derived the following equation for the calculation of $u_{2,s}$:

(23) $$\ln\left(1-u_{2,s}\right)+u_{2,s}+\chi_{1}u_{2,s}^{2}=\frac{V_{1}\rho }{M_{c}}\left(1-\frac{2M_{c}}{M_{n}}\right)u_{2,r}\left[{\left(\frac{u_{2,s}}{u_{2,r}}\right)}^{1/3}-\frac{1}{2}\left(\frac{u_{2,s}}{u_{2,r}}\right)\right]$$

where *χ*_1_ is the Flory’s interaction parameter of the polymer–solvent, *V*_1_ is the molar volume of the solvent and $u_{2,r}$ is the polymer volume fraction in the relaxed state. By multiplying the right-hand side term of Equation (23) with the correction parameter *g*, one can account for the effect of crosslinks undergoing some fluctuations, the effect of chain entanglements and intramolecular crosslinks that do not contribute to the network’s stiffness, as described by Tripathi and Tobita [20,52].

Accordingly, the mesh size of the network, *ξ*, can be calculated in terms of $u_{2,s}$, using the following equation [53,54,55]:

(24) $$\xi =u_{2,s}^{-\frac{1}{3}}{\left(\frac{\lambda C_{n}M_{c}}{MW_{m}}\right)}^{\frac{1}{2}}l$$

where *MW_m_* is the molecular weight of a repeating structural unit in a chain, *λ* is a backbone bond factor, *C_n_* is the Flory characteristic ratio and l is the length of the bond. In the case of hyaluronic acid, l is the bond length from glycosidic oxygen to glycosidic oxygen in a monosaccharide [56].

In the following section, the model predictions are compared with experimental measurements on plateau time, plateau storage modulus (*G′*) and average molecular weight between crosslinks (*M_c_*) for a tyramine modified hyaluronic acid crosslinkable solution [8]. Moreover, the calculated values for the polymer volume fraction in the swollen state ($u_{2,s}$) and mesh size (*ξ*) are reported for the same system. Finally, model predictions on the dynamic evolution of the storage modulus (*G′*) are compared with experimental measurements for the HA-Tyr system [8].

## 4. Comparison of Model Predictions with Experimental Results

The developed 4D kinetic Monte Carlo model detailed in Section 2 was used to simulate the crosslinking kinetic of a HA-Tyr solution in the presence of the HRP/H_2_O_2_ initiation system. The experimental results of Lee et al. (2008) [8] were used to estimate the kinetic model parameters and validate the MC model predictions. To reduce the computational time of the MC simulations, it was assumed that the initial sample of the HA-Tyr polymer chains was monodisperse. However, MC simulations carried out with an initial polydisperse sample of polymer yielded similar results.

The initial values of the kinetic rate constants appearing in the postulated kinetic mechanism (Equations (1)–(3) and (5)) were obtained from the literature. Thus, according to the reported values of the kinetic rate constants, the value of *k*_1_ varied in the range of (4.14∙10^5^–2∙10^7^) [28,57,58,59,60,61], and *k*_2_ and *k*_3_ varied in the range of (3.71∙10^3^–6.79∙10^6^) [58,59,60] and (1.45∙10^4^–4.56∙10^6^) [58,59,60,61], respectively, depending on the substrate type [59,60]. However, the numerical value of *k_2_* is generally an order of magnitude larger than *k*_3_ [59,60]. In the present study, the numerical values of the kinetic model parameters (i.e., *k*_1_, *k*_2_, *k*_3_, *k*_4_, *k*_5_) were estimated by fitting the predictions of a deterministic kinetic model (using the method of moments [62]) to the experimental measurements of Lee et al. [8] on the gelation onset time of a crosslinkable hyaluronic acid–tyramine aqueous solution under different crosslinking conditions (i.e., HRP and H_2_O_2_ concentrations). Subsequently, the estimated values of the kinetic rate constants (*k*_1_, *k*_2_, *k*_3_, *k*_4_, *k*_5_) were used in the present 4D stochastic Monte Carlo kinetic simulation model. The unknown value of the kinetic rate constant *k_6_* (see Chemical Reaction 6) was estimated by fitting the 4D MC predictions to the experimental measurements of Lee et al. [8] on the storage modulus *G′* and time required for *G′* to reach its plateau value.

Thus, for all the MC kinetic simulations, the following estimates of the kinetic rate constants were employed: *k_1_* = 10^7^ (L∙mol^−1^s^−1^), *k_2_* = 8∙10^5^ (L∙mol^−1^s^−1^), *k_3_* = 2.2∙10^4^ (L∙mol^−1^s^−1^), *k_4_* = 7.3∙10^2^ (L^2^mol^−2^s^−1^), *k_5_* = 10^12^ (L∙mol^−1^s^−1^) and *k_6_* = 50 (L∙mol^−1^s^−1^).

For all MC kinetic simulations, an original MATLAB software (see Figure 2 and Table 2) was employed. The computational time needed for an MC kinetic simulation largely depended on the initial number of the polymer chains in the sample. In Table 3, the effect of the initial number of polymer chains on the required computational time in sec is shown for the HA-Tyr [8]. All simulations were carried out with an Intel Core i7-6700 processor and 16 GB RAM. It is evident that, as the sample size increases, the computational effort largely increases. In particular, for an initial sample of 1,028,368 polymer chains, the computational time is equal to 20.233 h.

**Figure 4:**
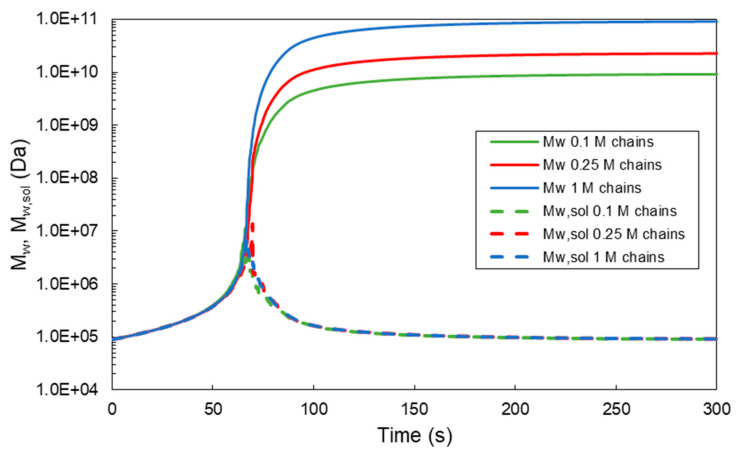
Effect of the initial number of polymer chains on the time evolution of the weight average molecular weight (*M_w_*) in the system and sol phase (*M_w,sol_*) for a HA-Tyr solution (i.e., 1.75% *w/v*, MW = 90 kDa, 0.124 units/mL HRP and 728-μM H_2_O_2_) [8].

In Figure 4, the effect of the initial number of polymer chains in the sample on the MC calculated overall weight average molecular weights in the system (*M_w_*) and in the solution phase (*M_w,sol_*) are depicted with respect to the crosslinking time. In particular, three different samples of approximately 0.1, 0.25 and 1 million polymer chains (e.g., 90 kDa) were selected to simulate the crosslinking kinetics of a HA-Tyr solution (i.e., 1.75% *w/v*) in the presence of 0.124 units/mL HRP and 728-μM H_2_O_2_ [8]. As can be clearly seen in all the simulated cases, the overall *M_w_* exhibits a very steep increase after the gelation onset time, followed by a plateau value (continuous green, red and blue lines in Figure 4). On the other hand, the *M_w,sol_* exhibits an initial increase up to the gelation onset time, followed by a subsequent decrease to a plateau value (see the broken green, red and blue lines in Figure 4). Note that the observed increase in *M_w_* is characteristic of the onset formation of a three-dimensional hydrogel network. It is also worth mentioning that the sample size does not affect the calculation of the gelation onset time (i.e., for different HRP and H_2_O_2_ concentrations), as well as the time evolution of *M_w,sol_*. However, the sample size does affect the final plateau values of *M_w_*. In fact, as the sample size increases, the MC calculated plateau value of *M_w_* increases. The last result is attributed to the fact that, when the sample size increases, the sum of the molecular weight of the final cluster of chains increases, and thus, the final molecular weight of the system increases.

**Figure 5:**
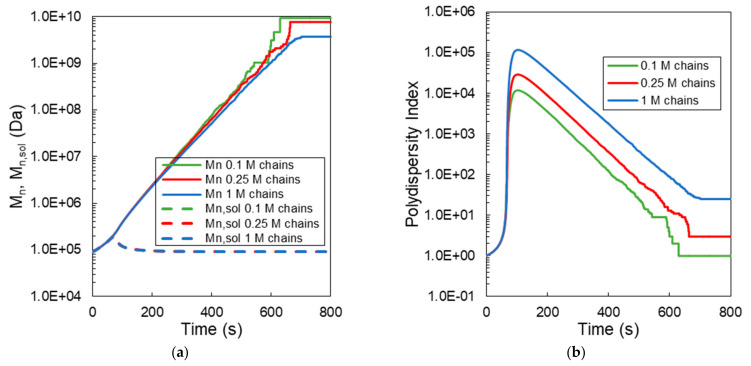
Effect of the initial number of polymer chains on the time evolution of the number of average molecular weights (*M_n_*) in the system and sol phase (*M_n,sol_*) (**a**) and Polydispersity Index in the system (**b**) for a HA-Tyr solution (i.e., 1.75% *w/v*, MW = 90 kDa, 0.124 units/mL HRP and 728-μM H_2_O_2_) [8].

In Figure 5, the time evolution of the overall number average molecular weight (*M_n_*) in the system and sol phase (*M_n,sol_*) (Figure 5a), as well on the overall polydispersity index in the system (PDI) (Figure 5b), are depicted for three different samples of polymer chains. As can be seen, the *M_n_* value increases with the crosslinking time. Note that the effect of sample size on the MC calculated values is less pronounced than that seen for *M_w_* in Figure 4. On the other hand, the time evolution of PDI after the gelation onset time does depend on the initial sample size due primarily to its effect on *M_w_*, as seen before. Finally, the effect of the sample size on the calculated *M_n,sol_* values appears to be insignificant (Figure 5a).

For the determination of the gelation onset time, we follow the time evolution of the weight average molecular weight in the solution. It is well-established that the time at which the *M_w_* shows a very steep increase (i.e., the value of *M_w_* increases by several orders of magnitude) characterizes the gelation onset time and the formation of a 3D polymer network. Thus, the gelation onset time is defined as the time at which the *M_w,sol_* attains a maximum. From that time on, the gel fraction starts increasing while the polymer fraction in the solution decreases as polymer chains from the solution are connected to the growing polymer network. Regarding the calculation of gel and sol fractions in the Monte Carlo-based methods, different approximations have been proposed in the literature [20,36,63]. In the present MC algorithm, the largest polymer chain in the simulated sample is treated as gel, while all the remaining polymer chains in the sample are considered to be in the solution phase [36,63]. Thus, the molecular weight and the mass fraction of the largest chain and the molecular weight and mass fraction of the remaining chains is bookkept during the whole simulation in order to find the maximum *M_w,sol_*.

**Figure 6:**
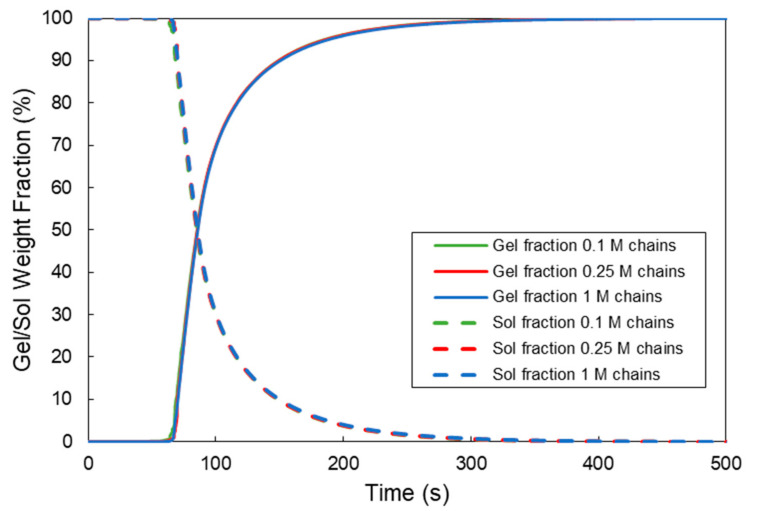
Effect of the initial sample size on the time evolution of the sol and gel mass fractions for a HA-Tyr solution (i.e., 1.75% *w/v*, MW = 90 kDa, 0.124 units/mL HRP and 728-μM H_2_O_2_) [8].

In Figure 6, the MC calculated gel and sol fractions in a Tyr-HA polymer solution (i.e., 1.75% *w/v*, MW = 90 kDa) in the presence of 0.1245 units/mL HRP and 728-μM H_2_O_2_ are depicted for the three different initial sample sizes. As can be seen, the initial number of polymer chains in the sample does not affect the MC calculated values of the gel and sol polymer fractions. Note that, until the gelation onset time, the values of the gel and sol polymer fractions remain constant and equal to 0% and 100%, respectively. Accordingly, after the gelation onset time, the gel fraction progressively increases to its final value (i.e., 100%), while the respective value for the sol fraction decreases to 0%, which marks the complete incorporation of all polymer chains into the network.

**Figure 7:**
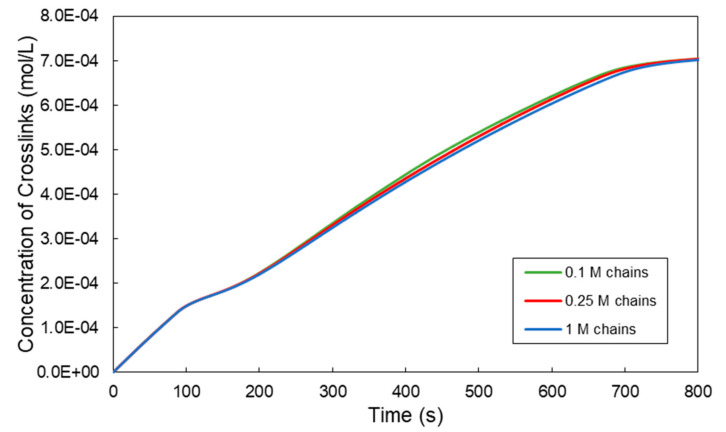
Effect of the initial sample size on the time evolution of the crosslinks concentration for a HA-Tyr solution (i.e., 1.75% *w/v*, MW = 90 kDa, 0.124 units/mL HRP and 728-μM H_2_O_2_) [8].

At this point, it should be pointed out that one of the main advantages of the present 4D Monte Carlo algorithm is that it can simulate the crosslinking kinetics and evolution of molecular properties and gel and sol fractions before and after the gelation onset time. Moreover, at any time during the simulation, it is possible to know all the molecular structural features of all polymer chains in the system (e.g., concentration of the residual phenol groups), including the crosslinks concentration. From the knowledge of the crosslinks concentration, one can calculate the dynamic evolution of the storage modulus of hydrogel, *G′*. In Figure 7, the time evolution of the crosslinks concentration is depicted for the crosslinkable Tyr-HA system under investigation. It is evident that the initial sample size does not have any significant effect on the crosslinks concentration.

Effect of HRP and H_2_O_2_ on the gelation onset time.

**Figure 8:**
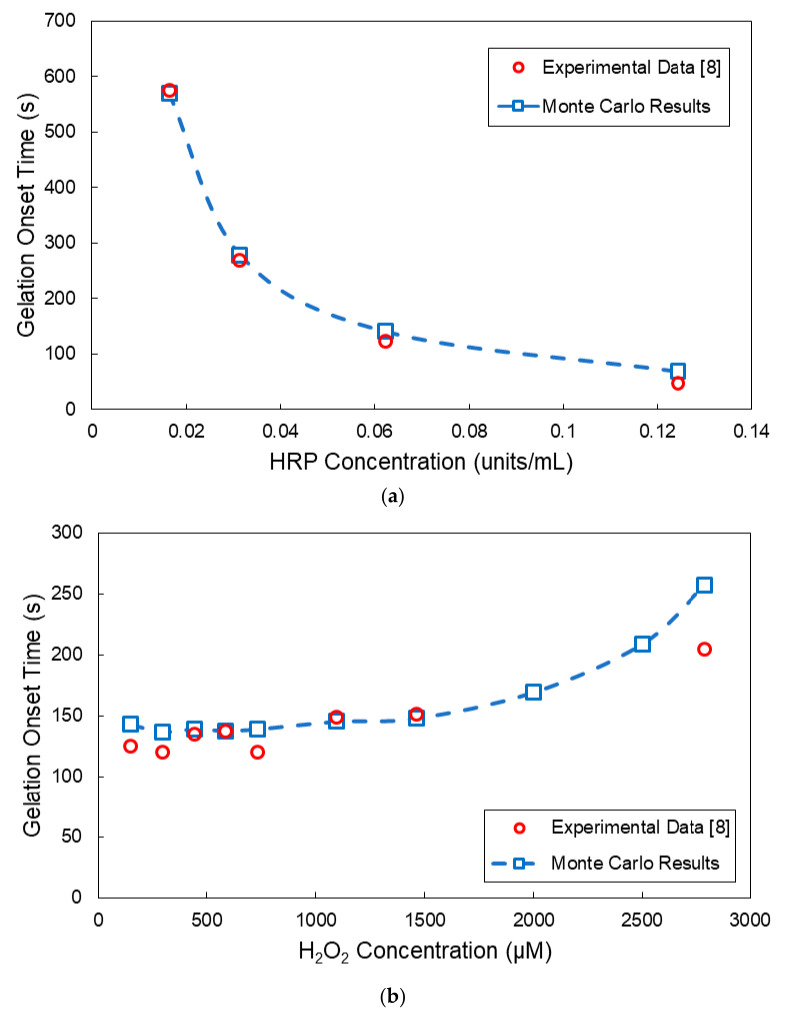
Comparison of the MC model results with reported experimental measurements on the gelation onset time for the HA-Tyr system (i.e., 1.75% *w/v*, MW = 90 kDa) [8]: (**a**) effect of the HRP concentration on the gelation onset time (H_2_O_2_ concentration: 728 μM) and (**b**) effect of the H_2_O_2_ concentration on the gelation onset time (HRP concentration: 0.062 units/mL).

**Figure 9:**
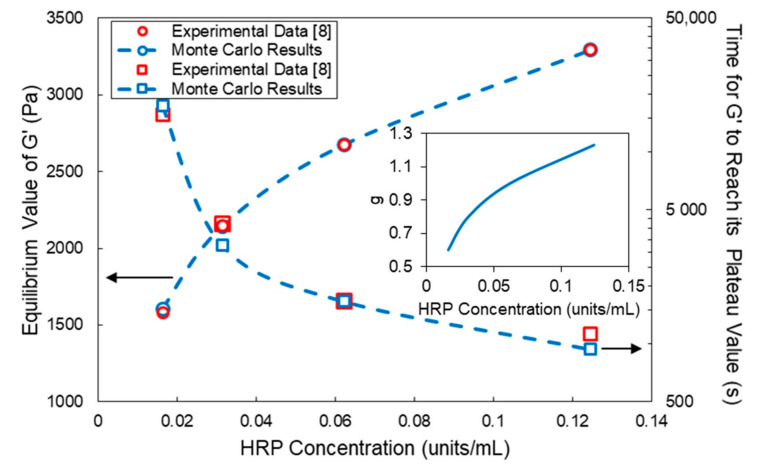
Effect of the HRP concentration on the *G′* equilibrium value and time required for *G′* to reach its plateau value for the HA-Tyr crosslinkable solution (i.e., 1.75% *w/v*, MW = 90 kDa and H_2_O_2_ concentration = 728 μM). Blue squares with a blue dashed line and blue circles with a blue dashed line represent the MC calculated values, and red circles and squares denote the respective experimental measurements of Lee et al. [8]. All MC simulations were conducted with an initial polymer chain population of ~10^6^.

**Figure 10:**
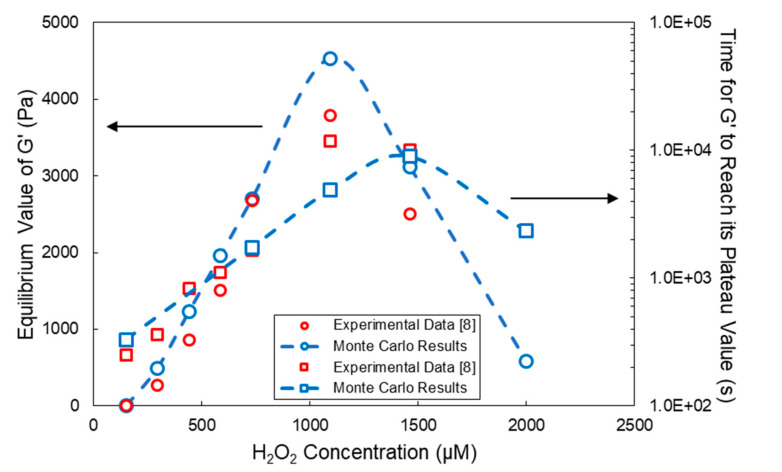
Effect of the H_2_O_2_ concentration on the *G′* equilibrium value and time required for *G′* to reach its plateau value for the HA-Tyr crosslinkable solution (i.e., 1.75% *w/v*, MW = 90 kDa and HRP concentration = 0.062 units/mL). Blue squares with a blue dashed line and blue circles with a blue dashed line represent the MC calculated values, and red circles and squares denote the respective experimental measurements of Lee et al. [8]. All MC simulations were conducted with an initial polymer chain population of ~10^6^.

**Figure 11:**
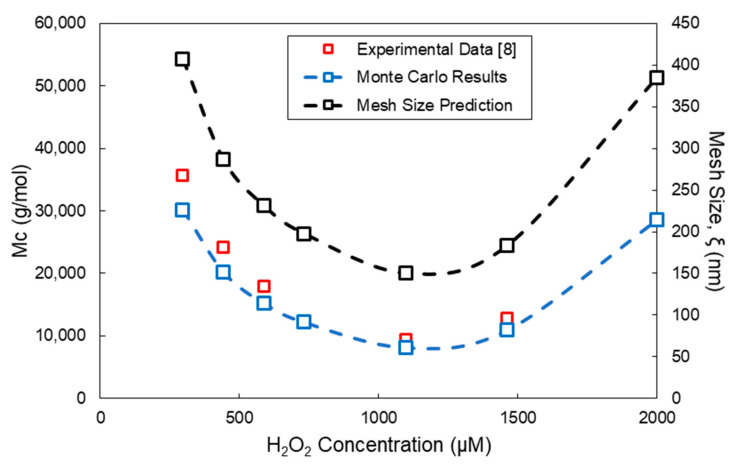
Effect of the H_2_O_2_ concentration on the molecular weight between crosslinks, *M_c_*, for the HA-Tyr crosslinkable solution (i.e., 1.75% *w/v*, MW = 90 kDa and HRP concentration = 0.062 units/mL). All MC simulations were conducted with an initial polymer chains population of ~ 10^6^.

In Figure 8, the effects of HRP and H_2_O_2_ on the gelation onset time for the HA-Tyr crosslinkable system are depicted. As can be seen, there is an excellent agreement between the MC model predictions and reported experimental results on the gelation onset time, especially in the case of the HRP variation, as shown in Figure 8a. In the case of the H_2_O_2_ variation (Figure 8b), the MC model predictions exhibit some small deviation from the corresponding experimental measurements, especially at very low and very high H_2_O_2_ concentrations. The observed differences can be attributed to (i) the accuracy of the experimental measurements at very small and high gelation times, (ii) the number of MC simulations performed for each selected H_2_O_2_ concentration, (iii) the sample size and (iv) the general validity of the postulated kinetic mechanism over an extended range of variation of the H_2_O_2_ concentration. Note that, as the initial sample size increases, the MC simulation results do exhibit a smaller run-to-run variability. Moreover, as the number of MC simulations per case increases, the calculated mean value of the gelation onset time exhibits a smaller deviation from the experimentally observed value. In the present study, the plotted results represent the average value of two MC simulations per case (Figure 8, Figure 9, Figure 10 and Figure 11). In all MC simulations, the initial sample size contained circa 10^6^ polymer chains.

Prediction of hydrogel viscoelastic and structural properties

Based on the theoretical developments discussed in Section 3 (Equations (17), (20) and (21)) and the MC calculation of the time variation of the crosslinks concentration (see Figure 7), the value of the equilibrium (plateau) storage modulus, *G′(ω)*, as well as the time required for the HA-Tyr crosslinkable solution to reach its plateau value were calculated. It should be noted that the 4D Monte Carlo model included a cyclization reaction (Equation (6)). Moreover, it was assumed that the equilibrium shear modulus *G_e_* could be approximated by the experimentally measured plateau value of *G′(ω)* at the applied low frequency ω (i.e., 1 Hz).

Note that all the 4D MC simulations were carried up to a max crosslinking time of 45,000 s that is well beyond the gelation onset time, to ensure that all the reactive polymer chains had reacted. Note that the time required for the storage modulus to reach its plateau value was estimated by monitoring the time-varying number of crosslinks per initial polymer chain. Thus, the reaction time at which the number of crosslinks per initial polymer chain had reached approximately 98% of its max theoretical value was taken as the time at which the storage modulus attained its plateau value. 

In Figure 9, the effect of the HRP concentration on the *G′* equilibrium (plateau) value and the time required for *G′* to reach its plateau value are depicted. In the inset diagram in Figure 9, the variation of the *g* factor in Equation (21) is depicted with respect to the HRP concentration. As can be seen, the value of the correction factor *g* increases with the HRP concentration, i.e., the number of effective crosslinks increases due to the increased number of chain entanglements (see Figure 3). Note that, as the HRP concentration increases, the time required for *G′* to reach its respective plateau value decreases, which is attributed to the increase in the net production rate of crosslinks with the HRP concentration. The MC results (blue circles with a blue dashed line and blue squares with a blue dashed line) shown in Figure 9 are in excellent agreement with the respective experimental (red circles and squares) measurements of Lee et al. [8]. It should be pointed out that, despite the increase in the net production rate of crosslinks with the HRP concentration, the maximum theoretical number of chemical crosslinks is not affected, for it depends on the total concentration of tyramine functional groups in the system and the rate of the enzyme deactivation reaction. Thus, the observed increase in the plateau value of *G′* was attributed to the increased number of chain entanglements at higher HRP concentrations effected by the fast crosslinking kinetics and consequential decrease in chain mobility. In the preset work, the postulated increase of chain entanglements with the HRP concentration was taken into account via the variation of the *g* factor in Equation (21) with the HRP concentration (see the inset diagram in Figure 9).

In Figure 10, the effect of the H_2_O_2_ concentration on the *G′* equilibrium (plateau) value (blue circles with a blue dashed line) and the time required for *G′* to reach its plateau value (blue squares with a blue dashed line) are depicted. The red discrete points show the respective experimental measurements [8]. As can be seen, as the H_2_O_2_ concentration increases, the equilibrium value of *G′* increases up to a maximum value. This is followed by a subsequent decrease in *G′* at higher H_2_O_2_ concentrations. Similarly, the time required for *G′* to reach its plateau value exhibits an initial increase with the H_2_O_2_ concentration, followed by a subsequent decrease at higher H_2_O_2_ concentrations. Note that the observed decrease in the values of the *G′* and plateau time at high H_2_O_2_ concentrations (i.e., above circa 1000 μM) is due to enzyme deactivation (Equation (4)) and the subsequent decrease of the respective rates of termination by combination (Equation (5)) and cyclization (Equation (6)) reactions.

It should be noted that, at very low (<200 μM) and high (<2000 μM) H_2_O_2_ concentrations, the MC calculated concentration of crosslinks, *v_c_*, is less than 1 (i.e., below its critical cut-off value). This means that the application of Equation (21) is not valid for the network correction term (1 − *ρ/(v_c_∙M_n_)*), accounting for loose chain ends, is negative and, thus, the calculated value of G_e_. It is apparent that the MC calculated results for *G′* at equilibrium and corresponding plateau time do deviate from the respective experimental values at low and high H_2_O_2_ concentrations, for the hydrogel network under those conditions is far from ideal [45,46]. Moreover, the postulated kinetic mechanism may not be applicable over such an extended range of variation of H_2_O_2_ concentration.

Using Equation (21) and the experimental measurements of Lee et al. [8] on the equilibrium storage modulus (*G′*), the molecular weight between crosslinks (*M_c_*) can be estimated for *g* = 1. Moreover, from the application of Equation (22) and the MC calculated values of the crosslinks concentration, *v_c_*, the M_C_ values can be calculated. In Figure 11, the experimentally determined values of M_c_ (red discrete points) are compared with the respective values of M_c_ calculated by the Monte Carlo method (blue squares with a blue dashed line) for different H_2_O_2_ concentrations. Note that, as the H_2_O_2_ concentration increases, the *M_c_* value initially decreases up to a minimum value, followed by a subsequent increase in *M_c_* at higher H_2_O_2_ concentrations. It is worth mentioning that the results of Figure 11 on the *M_c_* variation are in full agreement with the calculated values of *G′* at equilibrium and the calculated variation of *v_c_* with respect to the variation of the H_2_O_2_ concentration. Moreover, from the calculated values of M_c_ and Equations (23) and (24), the polymer volume fraction in the swollen state, *u_2,s_*, and the mesh size, *ξ*, can be calculated. In Figure 11, the variation of the mesh size, *ξ*, with respect to the H_2_O_2_ concentration is depicted (black squares with a black dashed line).

Regarding the prediction of the dynamic evolution of *G′* during the crosslinking of the HA-Tyr solution, there is a limited number of references in the open literature despite its apparent importance in tissue engineering and biomedical applications. In the present study, using the MC calculated values of the gel mass fraction, *w_g_*, and a linear approximation of the correction factor *g′* with respect to the concentration of crosslinks, *v_c_* (i.e., *g′* = 0.8426∙*v_c_* − 0.0693), the dynamic evolution of the storage modulus of the crosslinkable HA-Tyr system*, G′*(*t*), was calculated using the following equation:

(25) $$G^{\prime }\left(t\right)=g^{\prime }2v_{o}w_{g}kT=g^{\prime }2v_{c}w_{g}RT$$

**Figure 12:**
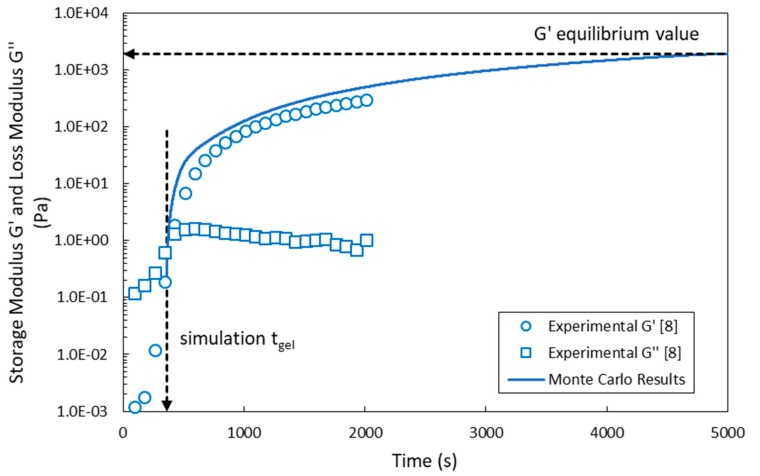
Comparison of MC results (blue line) with the reported experimental measurements (blue dots) on storage modulus during gelation for the HA-Tyr crosslinkable system [8]. The MC simulation results were obtained with an initial polymer chains population of ~10^6^. The vertical dash line marks the gelation onset time as predicted by the 4D MC algorithm. The horizontal dash line denotes the equilibrium value of *G′* as reported by Lee et al. [8]. The experimental measurements (blue squares) of the loss modulus (*G″*) are also depicted.

In Figure 12, the dynamic evolution of the storage modulus (*G′*) for the HA-Tyr crosslinkable solution system (i.e., 1.75% *w/v*, MW = 90 kDa, 728 μM of H_2_O_2_ and 0.025 units/mL HRP) is depicted [8]. The MC dynamic predictions of *G′* (blue continuous line) shown in Figure 12 are in good agreement with the respective experimental measurements (blue discrete points) of Lee et al. [8]. Note that the MC calculated values of *G′* are plotted after the gelation onset time at which the first gel fraction appears (i.e., the value of *w_g_* is different from zero). In Figure 12, the experimental measurements of loss modulus (*G″*) (blue discrete points) are also depicted [8]. Note that the numerical value of *g′*, calculated by the postulated linear approximation of *g′* in terms of crosslinks concentration at a time approximately equal to 5000 s (i.e., corresponding to the plateau value of *G′* [8]), was equal to 0.53. This value is equal to the product of network correction factor g (see inset Figure 9 for an HRP concentration of 0.025 units/mL) times the term *(*1 − *ρ/(v_c_∙M_n_)* (see Equation (21)); that is, *g′* = *g(*1 − *ρ/(v_c_∙M_n_) =* 0.73∙0.7274 *=* 0.53.

## 5. Conclusions

A four-dimensional Monte Carlo kinetic model was developed based on Gillespie’s stochastic algorithm [26,27] to simulate the crosslinking of polymer–phenol conjugate systems in the presence of HRP/H_2_O_2_ oxidation system. The model was applied to the investigation of gelation kinetics of HA-Tyr crosslinkable solutions. It was shown that the 4D MC model can predict the gelation onset time of the HA-Tyr solution under different crosslinking conditions (i.e., HRP and H_2_O_2_ concentrations). The MC kinetic model provided detailed on the molecular and structural properties of the HA-Tyr polymer chains (i.e., Number Chain Length Distribution, NCLD, bivariate Chain Length–Number of Crosslinks Distribution, *M_n_* and *M_w_* in the solution and gel phases, crosslinks concentration, gel mass fraction, etc.) before and after the gelation point. Based on the fundamental rubber elasticity theory and the MC calculated crosslinks concentration, the equilibrium storage modulus (*G′*) and the time required to reach the plateau value of *G′* were calculated for the HA-Tyr crosslinkable system. Model predictions were successfully compared with respective experimental measurements reported by Lee et al. [8]. Moreover, based on the MC results, the molecular weight between crosslinks (*M_c_*), as well as the hydrogel mesh size, *ξ*, were predicted for the same system. Note that the calculated values of *M_c_* and *ξ* can be further utilized to estimate the hydrogel diffusion coefficient of various solutes. Finally, it was shown that, using the present MC model, one can calculate the dynamic evolution of storage modulus for the HA-Tyr system. It is believed that the present 4D MC kinetic model can be used for the optimal design of a hydrogel having desired viscoelastic and molecular properties for as specific biomedical application.

## Figures and Tables

**Table 1:** Selected papers on HRP/H_2_O_2_ crosslinked polymer–phenol systems for biomedical applications.

Material	Authors
HA-Tyr	Kurisawa, Chung, Yang, Gao and Uyama 2005 [6]
Dextran-Tyr	Jin, Hiemstra, Zhong and Feijen 2007 [7]
HA-Tyr	Lee, Chung and Kurisawa 2008 [8]
Dextran-Tyr	Jin, Moreira Teixeira, Dijkstra, Zhong, Blitterswijk, Karperien and Feijen 2010 [9]
Carboxymethylcellulose-tyramine	Ogushi, Sakai and Kawakami 2007 [10]
Carboxymethylcellulose-phenolic hydroxyl groups (CMC-Ph)	Sakai, Ogushi and Kawakami 2009 [3]
Gelatin-hydroxyphenylpropionic acid (Gtn–HPA)	Wang, Chung, Chan and Kurisawa 2010 [11]
Dextran-tyramine (Dex-TA)/Hyaluronic acid-tyramine (HA-TA) conjugates	Wennink, Niederer, Bochynska, Teixeira, Karperien, Feijen and Dijkstra 2011 [12]
HA-Tyr	Ren, Gao, Kurisawa and Ying 2015 [13]
CMCH-Tyr	Bi, Liu, Kang, Zhuo and Jiang 2019 [14]

**Table 2:** Stochastic reaction rates for the 4D MC kinetic model.

Equation	Stochastic Reaction Rate	MC Simulation Algorithm
1	$R_{1}=k_{1,MC}N_{E}N_{H_{2}O_{2}}$	$N_{E}=N_{E}-1$ $N_{H_{2}O_{2}}=N_{H_{2}O_{2}}-1$ $N_{E_{I}}=N_{E_{I}}+1$
2	$R_{2}=k_{2,MC}N_{E_{I}}\overset{N_{RE}}{\underset{i=1}{\sum }}m_{i}R_{x_{i},m_{i},a_{i},c_{i}}$	$N_{E_{I}}=N_{E_{I}}-1$ Selection of $R_{x,m,a,c}$ $R_{x,m,a,c}\to R_{x,m-1,a,c}$ $N_{E_{II}}=N_{E_{II}}+1$
3	$R_{3}=k_{3,MC}N_{E_{II}}\overset{N_{RE}}{\underset{i=1}{\sum }}m_{i}R_{x_{i},m_{i},a_{i},c_{i}}$	$N_{E_{II}}=N_{E_{II}}-1$ Selection of $R_{x,m,a,c}$ $R_{x,m,a,c}\to R_{x,m-1,a,c}$ $N_{E}=N_{E}+1$
4	$R_{4}=\frac{1}{2}k_{4,MC}N_{E_{II}}N_{H_{2}O_{2}}\left(N_{H_{2}O_{2}}-1\right)$	$N_{E_{II}}=N_{E_{II}}-1$ $N_{H_{2}O_{2}}=N_{H_{2}O_{2}}-2$ $N_{E_{IV}}=N_{E_{IV}}+1$
5	$R_{5}=\frac{1}{2}k_{5,MC}\overset{N_{R}-1}{\underset{i=1}{\sum }}\left(a_{i}R_{x_{i},m_{i},a_{i},c_{i}}\overset{N_{R}}{\underset{l=i+1}{\sum }}a_{l}R_{y_{l},n_{l},b_{l},d_{l}}\right)$	Selection of $R_{x,m,a,c}$ Selection of $R_{y,n,b,d}$ Production of $R_{x+y,m+n,a+b-2,c+d+1}$ Removal of $R_{x,m,a,c}$ Removal of $R_{y,n,b,d}$
6	$R_{6}=k_{6,MC}\left(\frac{1}{2}a_{i}\left(a_{i}-1\right)G_{x,m,a,c}\right)$	Selection of $G_{x,m,a,c}$ $G_{x,m,a,c}\to G_{x,m,a-2,c+1}$

**Table 3:** Effect of the initial sample size on the computational time needed for the kinetic simulation of crosslinking of the HA-Tyr chains with 0.124 units/mL HRP and 728-μM H_2_O_2_ and PDI = 1 [8].

No of polymer chains	55,214	103,527	248,465	255,367	517,635	1,028,368
CPU in sec	173	586	4208	4371	16,657	72,839

## Data Availability

The data presented in this study are available on request from the corresponding author.

## Nomenclature

*C*	concentration of polymer in the solution
*C_n_*	Flory’s characteristic ratio
*E*	Horseradish Peroxidase (HRP)
*g*	network correction factor
*G*	relaxation modulus (Pa)
*G*′	storage modulus (Pa)
*G_e_*	equilibrium shear modulus treated by rubberlike elasticity theory (Pa)
*G_x,m,a,c_*	largest polymer chain with *x* monomeric units, *m* residual phenol groups, *a* activated phenol groups and *c* crosslinks
*k*	Boltzmann’s constant (J/K)
*k_j_*	kinetic rate constant of “*j*” reaction
*k_j,MC_*	stochastic kinetic rate constant of “*j*” reaction
*M_c_*	number average molecular weight between two crosslinks
*M_i_*	molecular weight of the “*i*th” polymer chain
*M_n,sol_*	number average molecular weight in the solution (g/mol)
*M_w_*	weight average molecular weight in the system (g/mol)
*MW_m_*	molecular weight of a repeating structural unit
*M_w,sol_*	weight average molecular weight in the solution (g/mol)
*N_A_*	Avogadro’s Number
NCLD	number chain length distribution
*N_E_*	the number of enzyme species E
*N_H2O2_*	total number of hydrogen peroxide species
*N_R_*	total number of polymer chains
*N_p_*	number of primary molecules before crosslinking
*N_RE_*	total number of reactions
*rand*1	randomly generated number uniformly distributed in the range of [0, 1]
*rand*2	randomly generated number uniformly distributed in the range of [0, 1]
$r_{E}^{2}$	mean square end-to-end distance of a strand
$\bar{r_{0}^{2}}$	mean square end-to-end distance of a non-constrained strand
*P_i_*	probability of reaction “*i*”
*R*	the universal gas constant (J∙mol^−1^K^−1^)
*R_j_*	the rate of the “*j*” chemical reaction
*T*	absolute temperature (K)
*u_2,r_*	polymer volume fraction at the relaxed state
*u_2,s_*	polymer volume fraction at the equilibrium swollen state
*V*	volume of the mixture
*V*_1_	molar volume of the solvent
*w_g_*	gel mass fraction
*x_i_*	degree of polymerization of the “*i*th” polymer chain
*X_c_*	total number of possible combinations of reactive species in a reaction
Greek Symbols	
Δ**t**	the time step between two reactions
*ν*	number of network chains or segments per unit volume (m^−3^)
*v_c_*	the number of moles of crosslinks per unit volume (mol∙m^−3^)
v_e_	number effective network chains or segments per unit volume (m^−3^)
*v_o_*	number of crosslinks per unit volume (m^−3^)
*λ*	backbone bond factor
*ξ*	mesh size of the network
*ρ*	polymer density (kg∙m^−3^)
*χ*_1_	Flory interaction parameter of the polymer-solvent
